# Understanding and management of gestational trophoblastic disease

**DOI:** 10.12688/f1000research.14953.1

**Published:** 2019-04-10

**Authors:** Fen Ning, Houmei Hou, Abraham N. Morse, Gendie E. Lash

**Affiliations:** 1 Guangzhou Institute of Pediatrics, Guangzhou Women and Children’s Medical Center, Guangzhou Medical University, Guangzhou, China

**Keywords:** gestational trophoblast disease, hydatidiform mole, choriocarcinoma

## Abstract

Gestational trophoblastic disease or neoplasia covers a spectrum of benign and malignant conditions arising from pregnancies with highly abnormal development of trophoblastic tissue. In this brief review, we discuss the different features of these different conditions and their origins and risk factors and introduce some of the more novel and controversial treatment options currently being explored.

## Introduction

Gestational trophoblastic disease (GTD) or neoplasia (GTN) covers a spectrum of benign and malignant conditions arising from malformed pregnancies. In this brief review, we discuss the different features of these different conditions and their origins and risk factors and introduce some of the more novel and controversial treatment options currently being explored.

## Normal trophoblast cell development

Fetal trophoblast cells are essential to facilitate embryo implantation into the uterus and are the major components of the placenta that ensure normal growth and development of the fetus ^1^. During placental development, the trophectoderm expands rapidly and starts to differentiate into both villous cytotrophoblasts (CTBs), which in turn differentiate into invasive extravillous trophoblast (EVT) and syncytiotrophoblast ^2, 3^. At the tips of anchoring villi, proliferating CTB generates columns to attach to the maternal decidua and differentiating EVT cells invade the maternal tissues, through the decidua and as far as the inner third of the myometrium ^2^. Within the trophoblast cell column, cells begin to express HLA-G as they differentiate from villous to extravillous trophoblast cells ^4^.

EVT cells invade the maternal tissues via two main routes, the interstitial and endovascular pathways, although there is also some evidence for endoglandular invasion ^5, 6^. Endovascular EVT cells invade the spiral arteries, retrograde to flow, plugging the artery openings so that for the first 10 weeks of gestation the developing embryo and placenta exist in a hypoxic environment ^5^. Interstitial EVT cells invade through the decidua and into the myometrium by breaking down the extracellular matrix and moving into the cleared spaces in a highly regulated manner. One of the main functions of invading interstitial EVT cells is in remodeling of the uterine spiral arteries, allowing adequate blood flow to the maternal–placental interface. Interstitial EVT cells also become terminally differentiated by forming non-invasive multinucleated giant cells, although the mechanism by which these cells are formed is not clear.

In addition to differentiating into EVT cells, CTB cells differentiate into the syncytiotrophoblast and continually fuse with it to replenish spent nuclei and organelles that are shed as apoptotic bodies known as syncytial knots, although the frequency with which this occurs is debated ^1, 6^. The syncytiotrophoblast is essential for gas and nutrient exchange across the placenta to and from the developing fetus.

## Gestational trophoblastic disease

**Figure 1.**
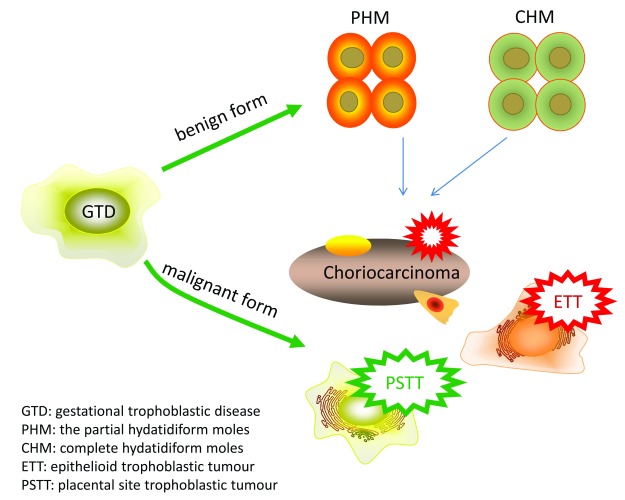
Schematic of the different benign and malignant forms of gestational trophoblastic diseases. Schematic of the different benign and malignant forms of gestational trophoblastic diseases.

GTD is a heterogeneous group of pregnancy-associated growths, often termed tumors, including choriocarcinoma, invasive mole, hydatidiform mole (partial and complete), epithelioid trophoblastic tumor (ETT), and placental site trophoblastic tumor (PSTT) ^7– 9^, that arise from placental villous and extravillous trophoblast cells ^10^. GTD has benign and malignant forms; the benign forms include partial hydatidiform moles (PHMs) and complete hydatidiform moles, whereas the malignant forms are choriocarcinoma (which can arise from hydatidiform mole, normal term pregnancy, ectopic pregnancy, or miscarriage), ETT, and PSTT ^11^ ( Figure 1 and Table 1). The major forms of GTD are choriocarcinoma and hydatidiform mole, and ETT and PSTT are relatively rare ^8^. GTD can occur weeks or years after any pregnancy but they occur most commonly after a molar pregnancy, which adds about 1 to 2% risk of further complete and partial mole ^8, 11, 12^.

**Table 1.** Clinical features of gestational trophoblastic disease ^13– 26^.

Gestational trophoblastic Disease	Clinical Manifestations	Incidence	Risk Factors	Cellular Origin
Choriocarcinoma	Irregular vaginal bleeding, cough, hemoptysis, headache, vomiting, convulsions, purple blue vaginal nodules, enlarged uterus, ovarian flavin cyst, intraperitoneal hemorrhage, high hCG levels	1 in 40,000 pregnancies	Age, deep myometrial invasion, tumor size, molar pregnancy, smoking, spontaneous miscarriage, ectopic pregnancy, site of metastases, disease duration, hCG level, stage	Villous trophoblast
Hydatidiform mole (complete)	Karyotype of 46XX, 46XY, diffuse villous enlargement with hydropic changes. varying atypia of trophoblast, absence of fetal tissue and villous capillaries, uterine enlarge , high hCG levels for gestational age, hypertension and hyperemesis gravidarum, abnormal bleeding, ovarian theca lutein cysts	0.57–1.1 per 1000 pregnancies	Age, ethnicity, and genetic basis, spontaneous miscarriage, nutrient, family history	Villous trophoblast
Hydatidiform mole (partial)	Karyotype of 69XXX, 69XXY or 69XYY, placental villi are focal edema and denatured that vary in size and shape, trophoblastic cells proliferate, fetal tissue is present, few full-term babies born, uteri are small for gestational age, low hCG level for gestational age, few medical complications	0.57–1.1 per 1000 pregnancies	Age, ethnicity, and genetic basis, spontaneous miscarriage, nutrient status, family history	Villous trophoblast
Epithelioid trophoblastic tumor	Abnormal vaginal bleeding	1 in 100,000 pregnancies	Stage, family history, deep invasion, tumor size	Intermediate trophoblast
Placental site trophoblastic tumor	Abnormal vaginal bleeding, trophoblastic infiltration confined to the endometrium and myometrium, hCG may be absent, resistant to chemotherapy	1 in 100,000 pregnancies	Age, deep invasion, tumor size, mitotic rate, disease stage, hCG level	Intermediate trophoblast

### Hydatidiform mole

Hydatidiform mole is a benign trophoblastic tumor ^27^ and accounts for the majority of GTD; about 80% of GTDs are hydatidiform moles ^13^. Hydatidiform mole is associated with abnormal gametogenesis and fertilization. The incidence varies around the world and is higher in Asia (~1 in 500) and the Middle East and Africa (~1 in 1000) than in Europe and North America (~1 in 1500) ^14, 28^. Risk factors include extremes of age, ethnicity, genetic basis, spontaneous miscarriage, and nutrient restriction ^8^. Women from 21 to 35 years of age have a lower risk of complete mole than women older than 35 years and younger than 21 years ^15^. Women with a history of prior spontaneous miscarriage have a two- to three-fold risk of molar pregnancy in comparison with the general population ^29^. Women with a history of molar pregnancy have a 10- to 20-fold risk of repeat molar pregnancy, and about 20% of patients will develop malignant transformation after evacuation of the mole.

**Figure 2.**
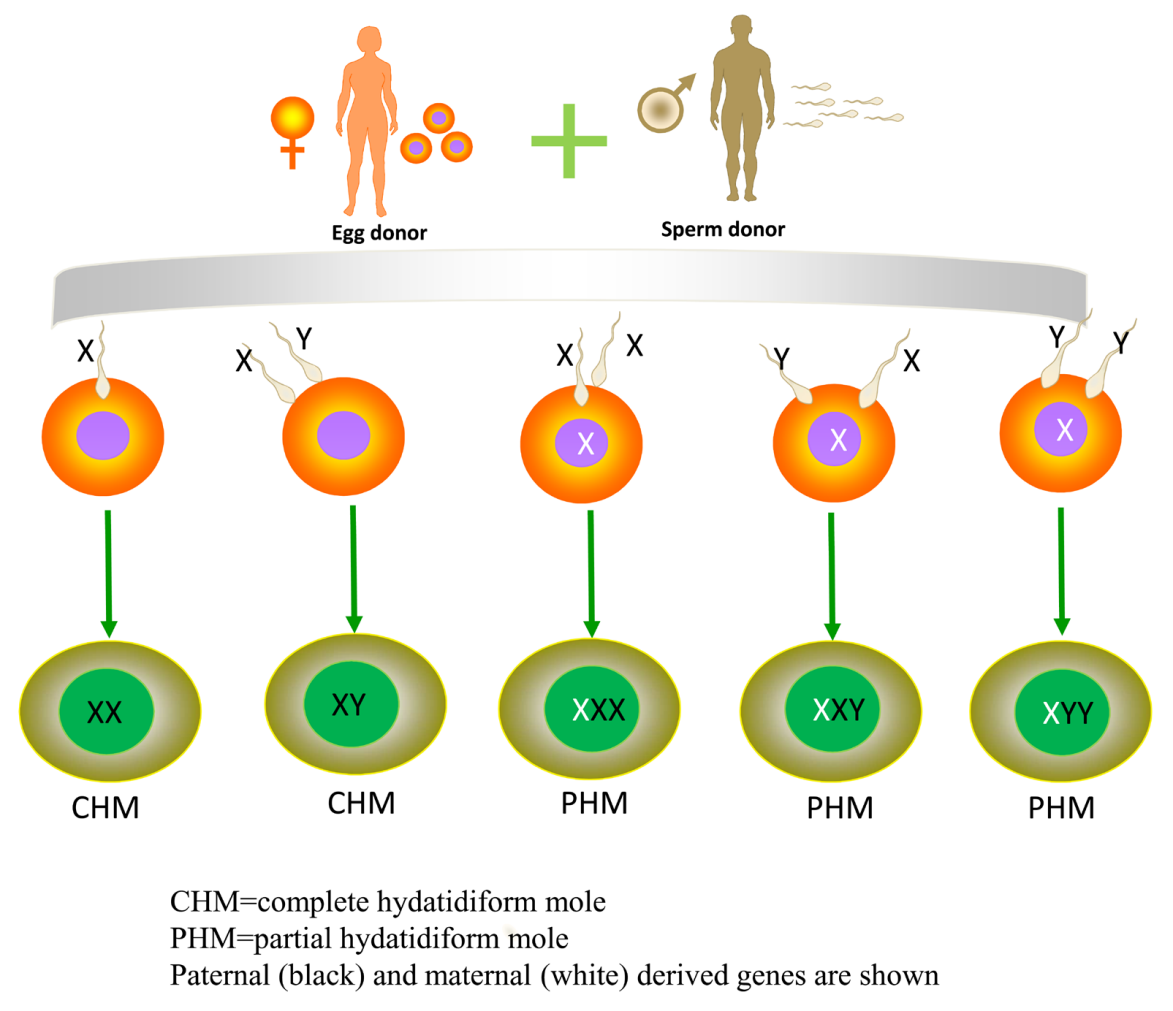
Schematic of the different karyotypes of complete and partial hydatidiform moles. Schematic of the different karyotypes of complete and partial hydatidiform moles.

Hydatidiform moles are edematous immature placentas which are broken down into complete and partial moles. A complete mole occurs when an empty ovum is fertilized by a sperm, about 90% of complete hydatidiform moles are 46XX which originate from duplication of the chromosomes of a haploid sperm and the other 10% are 46XY ( Figure 2) ^15^, and the chromosomes are paternally derived. Complete hydatidiform moles take on the appearance of a “bunch of grapes” which undergo diffuse villous enlargement with hydropic changes. The trophoblast has varying degrees of atypia and villous capillaries are absent. Fetal tissue or the embryo is absent in complete moles. In complete hydatidiform moles, the uterus is typically significantly enlarged for gestational age, and patients always have an elevated human chorionic gonadotropin (hCG) level for gestational age. Often, there can be early onset of medical complications such as pregnancy-induced hypertension, hyperthyroid, and hyperemesis gravidarum ^15, 30^. The most common presentation of molar pregnancy is abnormal vaginal bleeding during the first trimester and ovarian theca lutein cysts greater than 6 cm in diameter ^8, 31^.

A partial mole occurs when an empty ovum is fertilized by two sperm, the normal karyotype being 69XXX, 69XXY, or 69XYY, although a diploid karyotype may also exist ( Figure 2) ^15^. In PHMs, placental villi have focal edema and denatured areas of varying size and shape and pathological trophoblast cell proliferation. Fetal tissue or a recognizable embryo will be present. Most often, the fetus is not living, although occasionally there will be a small living fetus. Rarely, a term infant will be born.

### Choriocarcinoma

Choriocarcinomas are malignant trophoblastic tumors developing in the uterus from villous CTB cells ^15, 32^. About 50% of all choriocarcinomas arise from a complete molar gestation, 25% following a normal pregnancy, and 25% after a spontaneous miscarriage or ectopic pregnancy ^33^. Choriocarcinoma occurs in about 1 in 20,000 to 40,000 pregnancies in the United States and three to nine per 40,000 pregnancies in Southeast Asia and Japan ^34^. However, in all populations, the incidence rates of choriocarcinoma have declined over the past 30 years, although the precise incidence of choriocarcinoma may be under-reported because hemorrhage with biopsy precludes tissue diagnosis ^15, 16^.

Choriocarcinomas produce high levels of angiogenic growth factors and are able to remodel the uterine vasculature which can lead to hemorrhage ^35^. The clinical manifestation most often includes irregular vaginal bleeding, enlarged uterus, cough, hemoptysis, headache, and vomiting ^17, 36– 40^. Choriocarcinoma in the vagina can appear as purple/blue nodules and the uterus often becomes asymmetrically enlarged but not all women will present with all of these symptoms. In addition, intraperitoneal hemorrhage and high serum hCG levels exist ^32, 41^.

There are many potential predictive factors for choriocarcinoma. Rodabaugh *et al*. ^18^ found that a pretreatment hCG level of more than 100,000 mIU/mL, disease duration greater than 4 months from delivery, and the presence of liver or brain metastases were important predictors of outcome in patients with choriocarcinoma. The risk of choriocarcinoma is increased in women younger than 20 and in women older than 39 ^14^. Deep myometrial invasion, tumor size, tumor stage, and site of metastases also influence the outcome of patients with choriocarcinoma ^15, 32, 41^. Usually, choriocarcinoma occurs secondary to molar pregnancy, spontaneous miscarriage, or ectopic pregnancy but can also occur after full-term normal pregnancy ^8^.

## Epithelioid trophoblastic tumor and placental site trophoblastic tumor

ETT and PSTT are malignant trophoblastic tumors that arise from intermediate trophoblast cells of the placental bed after a full-term pregnancy or a non-molar miscarriage ^14, 19^. The incidence is about 1 in 100,000 pregnancies and ETT and PSTT represent just 0.2 to 2% of GTD cases but have the highest mortality rate ^19, 20, 42^. Unlike other forms of GTD, hCG may be absent in PSTT and it is fairly resistant to chemotherapy and the treatment is often complete hysterectomy, although more recently fertility-sparing surgery has been offered with limited success but it requires careful post-surgery monitoring to ensure that it has been curative ^14, 43^. Trophoblast cell infiltration is confined to the endometrium and myometrium in PSTT, and invasion is characterized by cells infiltrating the muscle fibers. These patients often present with lung metastases ^14, 16^. Advanced age (greater than 34 years), deep myometrial invasion, and tumor size have been associated with a worse outcome for patients ^19, 21, 44^.

## Treatment options for gestational trophoblastic disease

### Standard treatment options

Standard treatment options for GTD differ depending on the type and stage of disease and include chemotherapy, dilatation and curettage (D&C), or hysterectomy or a combination of these. In general, D&C is used for molar pregnancy and where a woman wishes to retain her fertility; however, careful post-treatment monitoring is required to ensure no recurrence of disease. For more severe and chemoresistant disease and when fertility preservation is not a concern, hysterectomy is the more common option, particularly if there are no distant metastases. Some types of GTD respond well to chemotherapy, either single or combined therapy; however, chemotherapy is not effective for all types of disease. Common chemotherapeutic agents include methotrexate, actinomycin D, etoposide, cyclophosphamide, vincristine, and cisplatin. There is a high risk of metastatic spread of some types of GTD and therefore a combination treatment modality of hysterectomy and chemotherapy is often employed. Careful β-hCG or human placental lactogen testing or both are required to ensure efficacy of any treatment modality. The majority of GTDs are treatable with one of the above common options; however, some more novel and controversial treatment modalities have recently been introduced and will be discussed further here.

### Prophylactic chemotherapy

One aspect of the treatment of GTD that is still controversial is whether to initiate prophylactic chemotherapy in a subpopulation of women with hydatidiform mole who are at high risk of persistence rather than following their hCG levels until they achieve the criteria for declaring no evidence of disease or meet the definition of persistent GTD. The idea is to reduce the need for more intense chemotherapy regimens in a smaller group of women by administering a more modest regimen to increase the chance of complete resolution. Several non-randomized trials demonstrated impressive reductions in the risk of recurrent/persistent disease. For example, in Korea, Kim *et al*. reported on 262 patients who were identified as having high-risk hydatidiform mole ^45^. Fifty (19%) received prophylactic chemotherapy and the remaining 216 patients served as controls. There were no cases of persistent GTD in the 59 patients who received prophylactic chemotherapy, but 59% of the control group developed persistent GTD ^45^.

In a randomized trial of a single dose of actinomycin D, 18% of the high-risk patients who received prophylactic chemotherapy and 34% of the high-risk patients who did not developed recurrent GTD (risk ratio [RR] = 0.54, 95% confidence interval [CI] = 0.35–0.82, number needed to treat = 7). Adverse events were similar in the two groups, and progression was not associated with increased disease severity in the chemotherapy group. Also, costs were lower with the prophylactic chemotherapy strategy ^46^.

A recent Cochrane review identified only three randomized trials, including the study of actinomycin D summarized above. The combined studies demonstrated a reduced risk of GTD (RR 0.37, 95% CI 0.24 to 0.57) but the Cochrane authors judged two of the three studies to be of low quality. The Cochrane authors concluded that prophylactic chemotherapy might reduce the risk of progression to GTD in women at high risk, but the strength of the conclusion is limited by the poor quality of the studies. Concerns about increased drug resistance, delays in treatment of GTD, and toxic side effects remain, so they conclude: “it is not possible to strongly recommend the practice” ^47^.

### Second dilatation and curettage

Another area where there is some divergence of typical practice in the treatment of GTD is whether to perform a second uterine curettage when a patient’s hCG trend is non-reassuring after initial diagnosis and evacuation of a molar pregnancy. The classic teaching was that owing to the risk of life-threatening hemorrhage or uterine perforation (or both), a second D&C should not be performed.

However, in some centers with substantial experience in treating GTD, second uterine evacuation appears to prevent the development of persistent disease and reduce the requirement for chemotherapy ^48^. In the GOG 242 trial, 60 women with a first diagnosis of low-risk GTD underwent a second uterine curettage and 24 (40%) subsequently experienced complete resolution of disease without the need for chemotherapy. The study noted that no patient with an hCG level of greater than 100,000 mIU/mL was cured and no patient with an International Federation of Gynecology and Obstetrics/World Health Organization (FIGO/WHO) score of more than 4 was cured. Success rate also appeared to be lower in women younger than 19 and older than 40. No surgical complications were reported ^49^.

### Selective uterine surgery

Women with chemoresistant disease typically are counseled to consider hysterectomy; however, many women in this position wish to retain their fertility. A review of the literature reports on women with PSTT who received fertility-saving treatment. Of 11 women who had laparotomy with uterine preservation, six patients had what was considered a successful procedure and the remaining five required total hysterectomy ^43^. Therefore, although this therapy may be effective in about 50% of cases, careful monitoring of excision margins and further disease progression are required. However, in the case of PSTT, hCG monitoring is not useful and a better marker of disease presence is human placental lactogen. In addition, studies on the effectiveness of these treatments for saving fertility have yet to be conducted.
